# Comparison of proteomic and genomic analyses of vaccine cell lines shows men receiving prostate gvax immunotherapy develop increased humoral responses to common cell line proteins relative to mutated proteins

**DOI:** 10.1186/2051-1426-2-S3-P49

**Published:** 2014-11-06

**Authors:** Tyler Hulett, Sachin Puri, Brendan Curti, Walter J Urba, Bernard A Fox

**Affiliations:** 1 Oregon Health & Science University, Portland, OR, USA; 2 Earle A. Chiles Research Institute, Providence Cancer Center, Portland, OR, USA; 3 Robert W. Franz Cancer Research Center at the Earle A. Chiles Research Institute, Providence Cancer Center, Portland, OR, USA

## 

Recent work by Rosenberg, et. al. demonstrates the role of mutated epitopes in natural anti-cancer adaptive immunity. This has led some to hypothesize that cancer vaccines may need to induce or augment immunity against mutated and potentially patient-specific epitopes to achieve therapeutic efficacy. In the case of whole-cell cancer vaccines, this could happen either indirectly through epitope spreading, or directly against 'hotspot' mutation sites on oncogenes. The purpose of this study is to determine whether patients who received an irradiated GM-CSF-secreting whole-cell prostate cancer vaccine, GVAX, preferentially developed humoral immune responses against mutated proteins. Archived pre-treatment and week 11 serum from patients enrolled on an investigator-initiated combination immunotherapy trial were assessed using protein arrays (ProtoArray, Invitrogen). Antibody responses that were increased in the week 11 sera were considered increased as a result of immunotherapy. Next we utilized reported liquid chromatography tandem mass spectrometry (LC-MS/MS) proteomics and whole-exome sequencing data for LNCaP and PC3, the two cell lines contained in prostate GVAX, to assess whether the targets of elevated antibody responses detected by protein array were directed preferentially against the mutated proteins contained in the vaccine. Of the 7554 different human proteins included in our analysis, a mean of 63 candidate responses were identified for each patient (SD = 43). These candidate responses were then compared to published non-synonymous mutations in LNCaP and PC3, published LC-MS/MS proteomics on the protein content of LNCaP and PC3, and randomized lists of proteins on the array [1-3]. Preliminary analysis suggests that there was no increase in antibody responses above background against proteins with published cell-line specific mutations. However, the number of antibody responses was increased against proteins identified by mass spectrometry (p = 0.017). This result suggests that when GM-CSF secreting whole-cell vaccine is not combined with other immunotherapies, vaccine alone may be insufficient to induce or augment immune responses against mutated epitopes.

Supported by The Prostate Cancer Foundation, DAMD PC02009, Robert W. Franz, the Chiles Foundation, ARCS Foundation: Portland, and the OCTRI, OSLER TL1 award.
